# Effects of atmospheric pressure change during flight on insulin pump delivery and glycaemic control of pilots with insulin-treated diabetes: an in vitro simulation and a retrospective observational real-world study

**DOI:** 10.1007/s00125-024-06295-1

**Published:** 2024-11-04

**Authors:** Gillian L. Garden, Ka Siu Fan, Megan Paterson, Fariba Shojaee-Moradie, Monique Borg Inguanez, Antonios Manoli, Victoria Edwards, Vivienne Lee, Brian M. Frier, Ewan J. Hutchison, Declan Maher, Chantal Mathieu, Stuart J. Mitchell, Simon R. Heller, Graham A. Roberts, Kenneth M. Shaw, Gerd Koehler, Julia K. Mader, Bruce R. King, David L. Russell-Jones, EASA Diabetes Consortium, Chantal Mathieu, David Russell-Jones, E. Marelise W. Eekhoff, Ewan Hutchison, Fariba Shojaee-Moradie, Felice Strollo, Gerd Köhler, Graham Roberts, Julia Mader, Monika Cigler, Renald Mecani, Richard Helsdingen, Stuart Mitchell, Thomas Pieber

**Affiliations:** 1 Faculty of Health and Medical Science, University of Surrey, Guildford, UK; 2 Centre for Endocrinology and Diabetes Research, Royal Surrey NHS Foundation Trust, Egerton Road, Guildford, UK; 3 School of Medicine and Public Health, University of Newcastle, Newcastle, NSW Australia; 4 Department of Paediatric Endocrinology and Diabetes, John Hunter Children’s Hospital, New Lambton Heights, NSW Australia; 5 Hunter Medical Research Institute, Newcastle, NSW Australia; 6 Department of Statistics and Operations Research, University of Malta, Msida, Malta; 7 Division of Endocrinology and Diabetology, Department of Internal Medicine, Medical University of Graz, Graz, Austria; 8 QinetiQ, Cody Technology Park, Farnborough, UK; 9 The Queen’s Medical Research Institute, University of Edinburgh, Edinburgh, UK; 10 Medical Department, Civil Aviation Authority, Aviation House, Crawley, UK; 11 Irish Aviation Authority, Dublin, Ireland; 12 Department of Endocrinology, KU Leuven, Leuven, Belgium; 13 Division of Clinical Medicine, School of Medicine and Population Health, University of Sheffield Medical School, Sheffield, UK; 14 CRF-C University College Cork, HRB Clinical Research Facility Cork, Mercy University Hospital, Cork, Ireland; 15 Diabetes Research Group, Swansea University, Swansea, UK; 16 Faculty of Science and Health, University of Portsmouth, Portsmouth, UK; 17 Austrocontrol, Vienna, Austria

**Keywords:** Aviation, Boyle’s law, Continuous subcutaneous insulin infusion, Henry’s law, Hypobaric, Hypoglycaemia, Insulin pump, Insulin-treated diabetes, Pilots, Safety

## Abstract

**Aims/hypothesis:**

Glycaemic control and clinical outcomes in diabetes are improved by continuous subcutaneous insulin infusion (CSII). Atmospheric pressure changes during flights may affect insulin delivery from pumps and cause unintended metabolic consequences, including hypoglycaemia, in people with type 1 diabetes. The present report evaluates both hypobaric flight simulation and real-world data in pilots using insulin pumps while flying.

**Methods:**

In the flight simulation part of this study, an in vitro study of insulin pumps was conducted in a hypobaric chamber, de-pressurised to 550 mmHg to mimic the atmospheric pressure changes in airliner cabins during commercial flights. Insulin delivery rates and bubble formation were recorded for standard flight protocol. Insulin infusion sets, without pumps, were tested in a simulated rapid decompression scenario. The real-world observational study was a 7.5-year retrospective cohort study in which pre- and in-flight self-monitored blood glucose (SMBG) values were monitored in pilots with insulin-treated diabetes. Commercial and private pilots granted a medical certificate to fly within the European Union Aviation Safety Agency approved protocol and receiving insulin either by pump or multiple daily injections (MDI) were included.

**Results:**

In the flight simulation study, full cartridges over-delivered 0.60 U of insulin during a 20 min ascent and under-delivered by 0.51 U during descent compared with ground-level performance. During emergency rapid decompression, 5.6 U of excess insulin was delivered. In the real-world study, seven pilots using CSII recorded 4656 SMBG values during 2345 h of flying across 1081 flights. Only 33 (0.7%) values were outside an acceptable safe range (5.0–15.0 mmol/l [90–270 mg/dl]). No clinically significant fall in the median SMBG concentration was observed after aircraft ascent and no in-flight SMBG values were within the hypoglycaemic range (<4.0 mmol/l [<72 mg/dl]). Compared with pilots receiving MDI therapy, pilots using CSII recorded more SMBG values within the acceptable range (99.3% vs 97.5%), fewer values in the low red range (0.02% vs 0.1%), fewer in-flight out-of-range values (0.2% vs 1.3%) and maintained stricter glycaemic control during flight.

**Conclusions/interpretation:**

Ambient pressure reduction during simulated flights results in bubble formation and expansion within insulin cartridges. This causes unintended delivery of small insulin doses independent of pre-determined delivery rates and represents the maximum amount of insulin that could be delivered and retracted. However, in vivo, pilots using CSII in-flight did not experience a fall in blood glucose or episodes of hypoglycaemia during these atmospheric pressure changes and the use of insulin pumps can be endorsed in view of their clinical benefits.

**Graphical Abstract:**

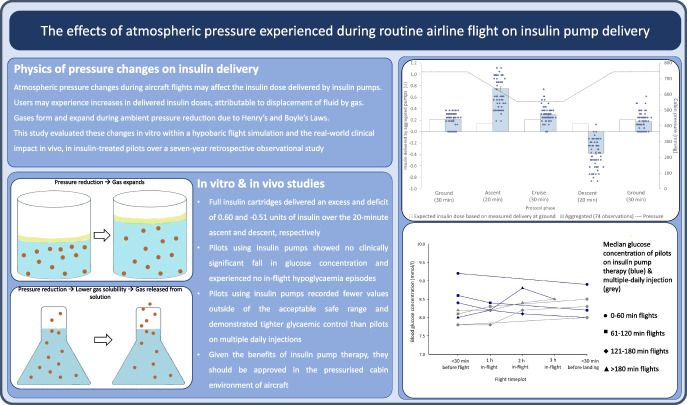

**Supplementary Information:**

The online version contains peer-reviewed but unedited supplementary material available at 10.1007/s00125-024-06295-1.


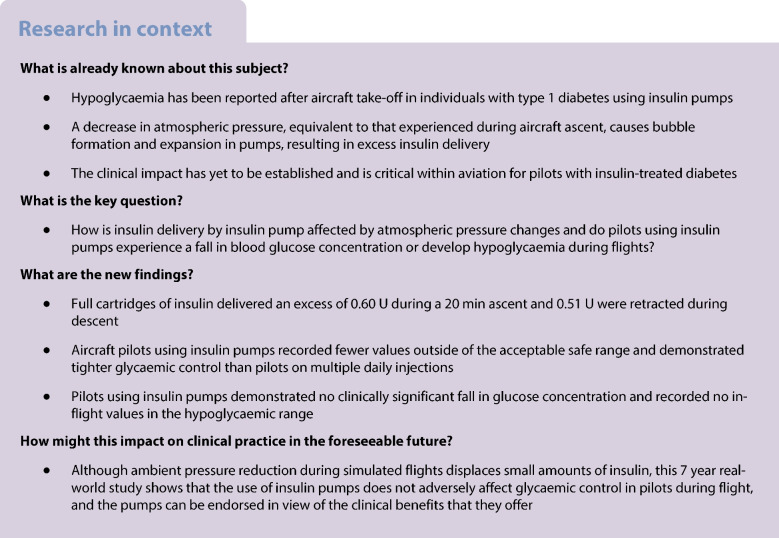


## Introduction

Technological advances in glucose monitoring and insulin delivery are enhancing the modern therapeutic management of diabetes. Continuous subcutaneous insulin infusion (CSII), using an insulin pump, delivers insulin effectively and safely and is associated with better glycaemic control and clinical outcomes [1, 2]. With diabetes becoming more prevalent, the number of people flying with insulin-treated diabetes is increasing [3] and consequently the number of both pilots and air passengers treated with CSII is also rising. However, despite many years of clinical use, the potential effects of ambient pressure changes on insulin pumps, with the consequential over- or under-delivery of insulin, are not well-described. Predictable ambient pressure changes during flight ascent and decent could have significant implications for glycaemic stability in pilots and their passengers.

The cruise altitude of a commercial aircraft is usually between 10,000 and 13,000 m (~30,000–42,000 feet). Commercial aircraft are normally pressurised to maintain a cabin pressure of approximately 560 mmHg, equivalent to an altitude of 2438 m (8000 feet) [4, 5]. Aanderud and Hansen observed that a decrease in cabin pressure, as experienced during aircraft ascent, causes an insulin pump to deliver more insulin than the pre-determined infusion rate [6]. King et al subsequently reported that the volume of insulin that was either over- or under-delivered could be predicted for a given change in ambient pressure by using data from studies of small pressure vessels [7]. This resulted from two independent processes. First, as dictated by Henry’s law, air that is dissolved in a solution moves out or degasifies when pressure decreases and the formation of air bubbles displaces insulin from the pump cartridge [8]. Second, in accordance with Boyle’s law, air bubbles that are already present inside the pump insulin cartridge expand as pressure decreases [9]. These phenomena were thought to explain some episodes of hypoglycaemia occurring 60–105 min after take-off, in both adults and children receiving CSII [7, 10, 11]. If this was experienced by a pilot of an aircraft, an unexpected additional bolus of insulin might cause hypoglycaemia and potential incapacitation. These studies have important implications for the operational protocols employed by countries that allow insulin-treated pilots to fly commercial aircraft.

In 2012, the UK Civil Aviation Authority (CAA) established a protocol to allow insulin-treated pilots to safely fly commercial and private aircraft. The Republic of Ireland and Austria joined the protocol in 2015 and 2016, respectively, utilising Part ARA.MED.330 of the EU Aircrew Regulation, enabling more than 130 insulin-treated pilots to fly since its inception [12]. Commercial airline pilots with insulin-treated diabetes granted a class 1 medical certificate and private pilots granted a class 2 medical certificate or a light aircraft pilot license (LAPL) must adhere to this protocol.

This is a two-part study involving both flight simulation and a real-world observational study that aimed to definitively evaluate the effects of pressure changes during flights on modern insulin pumps.

## Methods

### Flight simulation study

**Fig. 1:**
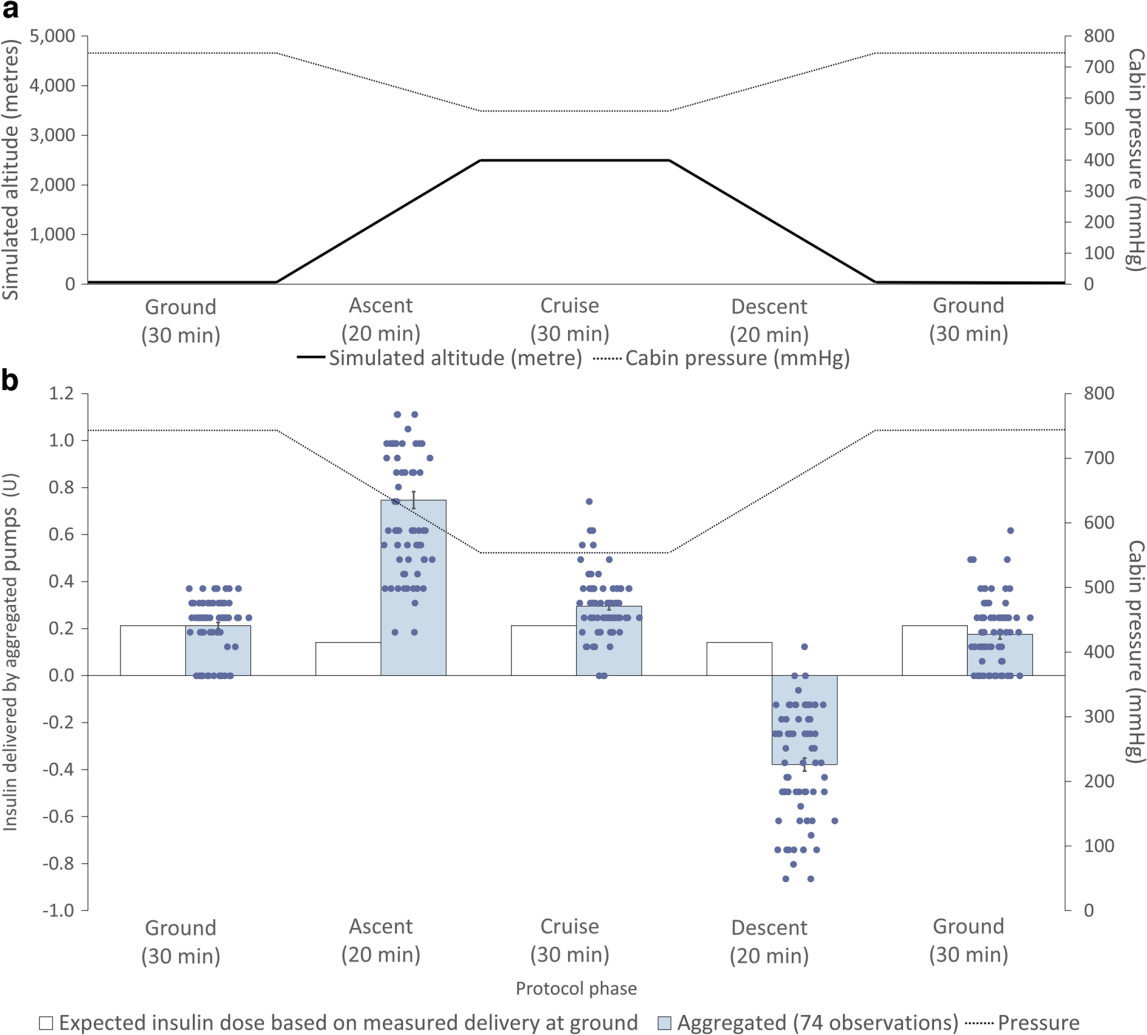
Illustration of the performance of insulin pumps in relation to the flight simulation protocol, altitude and cabin ambient pressures. (**a**) The different phases of the standard flight protocol, with the solid line representing the altitude in feet (scaled to the primary vertical axis) and the dotted line representing the ambient atmospheric pressure in mmHg (scaled to the secondary axis). (**b**) The aggregated insulin delivery of all pumps (blue, 74 observations) during phases of standard simulated flight protocol, against the measured ground-level basal infusion performance (white) of 0.6 U/h

**Fig. 2:**
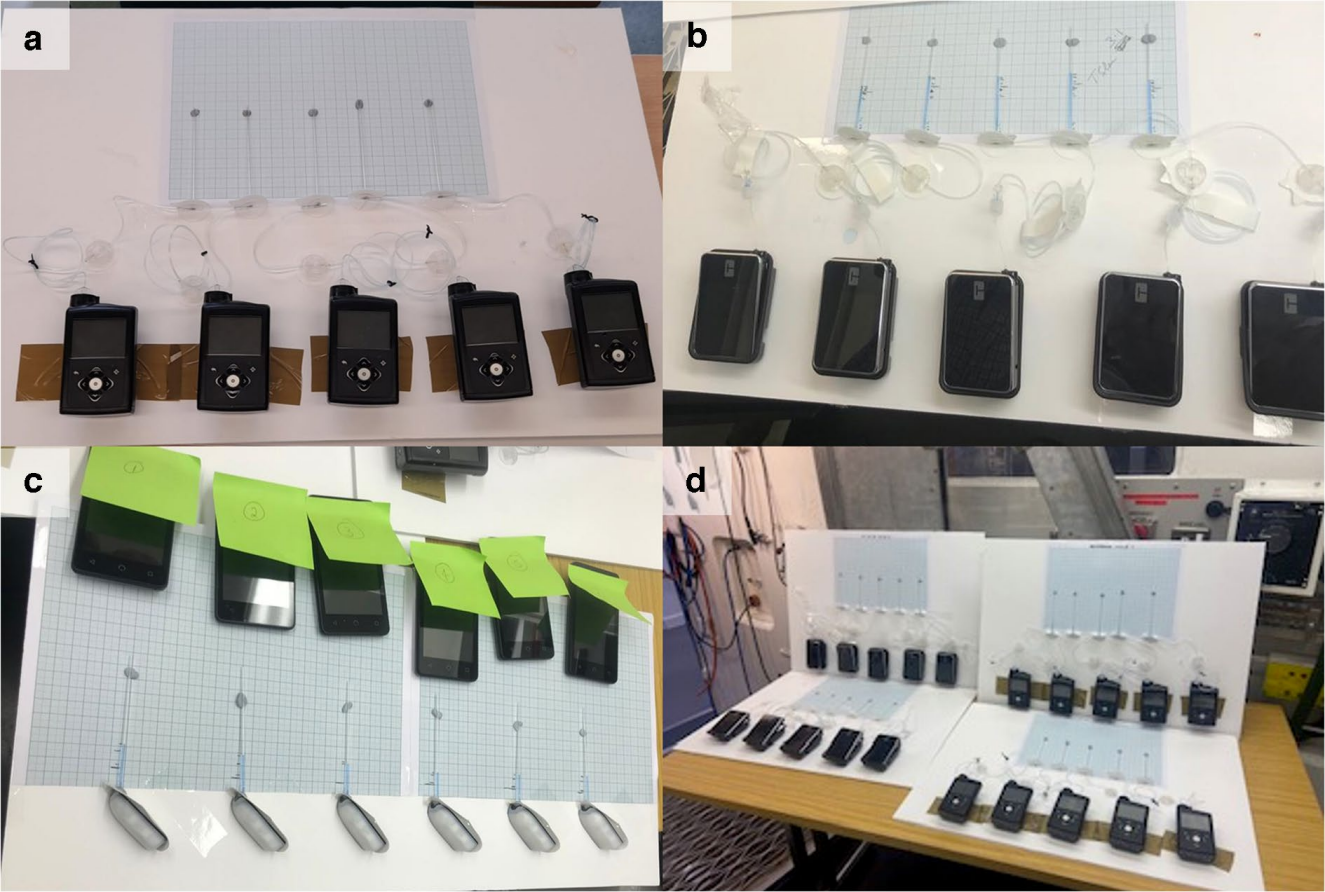
Experimental setup of each insulin pump. (**a**) Medtronic Minimed 780G insulin pump. (**b**) Tandem t:slim X2 insulin pump. (**c**) Insulet Omnipod DASH insulin pump. (**d**) Setup of insulin pumps and measurement grid paper within the hypobaric chamber

A hypobaric chamber (QinetiQ Hypobaric Research Facility, UK) was used to simulate the in-flight cabin pressures of a commercial airliner (Fig. 1a). Modern insulin pumps were studied with their cannulas open so that the effect of ambient pressure changes applied to the cannula, infusion line and cartridge could be assessed. Insulin pumps included Medtronic MiniMed 780G (Medtronic, USA), Tandem t:slim X2 (Tandem Diabetes Care, USA) and Omnipod DASH Insulin Management System (Insulet, USA). Insulin cartridges (Novo Nordisk, Denmark) were spiked with 8% vol./vol. blue food dye, with Omnipod DASH pumps using 2 ml cartridges and Medtronic 780G and Tandem t:slim X2 using 3 ml. Full pumps were connected to their infusion sets and inserted into plastic capillary tubes. The Omnipod DASH pump does not require an infusion set and its cannula tip was inserted directly into capillary tubes [7, 13]. All joints were connected using superglue (Loctite, Ireland) and Blu-Tack (Bostik, Ireland) to prevent leakage and were mounted against grid paper (Fig. 2). The pumps were set to infuse basal insulin at 0.6 U/h, equivalent to 6 µl/h, to represent both adult and paediatric use.

#### Standard flight simulation protocol

The protocol was as follows: 30 min on ground (750 mmHg); 20 min ascent to 2500 m (8200 feet) altitude (equivalent to 550 mmHg, to which commercial airliner cabins are pressurised); 30 min cruise (550 mmHg); 20 min descent to ground (750 mmHg); and then 30 min on ground. As cabin pressures vary with flight patterns and aircraft, we simulated gradual ascents and descents between 2500 m (8200 feet) and ground to reflect the extremes of normal operation [4]. The study and priming of pumps/lines were conducted on the ground, at 20°C. All insulin cartridges, pumps and infusion sets were allowed to equilibrate to ambient pressure and temperature within the chamber, prior to commencing the study. Measurements were taken at the beginning of each flight phase and a total of 26 pumps were studied with triplicate repeat flight simulations.

#### Rapid decompression–recompression protocol

Insulin pumps were not licensed for rapid decompression, so only the infusion sets and reservoirs were used to evaluate fluid movement and bubble formation. This protocol represents an example of flight manoeuvres following rapid decompression events. This simulates events such as window or door failures at 12,000 m (40,000 feet): 30 min at ground level (750 mmHg); 20 min ascent (550 mmHg); 5 min cruise; and then rapid decompression by dropping the pressure to 120 mmHg (approximately 12,000 m [40,000 feet equivalent]) over 10 s. The pressure was held at 120 mmHg for 5 min, then gradually raised to 260 mmHg (approximately 8000 m [25,000 feet equivalent]) over 10 s to mimic emergency flying response, then three further minutes of descent to 3800 m (12,000 feet) pressure-altitude (480 mmHg), followed by a 20 min descent to the ground.

#### Flight simulation study statistical analysis

The performance of each flight phase was compared against the measured performance at ground level to standardise findings and account for measurement errors. ANOVA was conducted, with *p*<0.05 considered statistically significant. Leakages and occlusions were excluded from the analysis.

### Real-world observational study

This retrospective observational study examined self-monitored capillary blood glucose (SMBG) values collected by pilots with insulin-treated diabetes between May 2012 and December 2019. Pilots working within the UK, Ireland and Austria, certified under the European Union Aviation Safety Agency (EASA) diabetes protocol, were invited to participate in this study. The inclusion criteria were pilots of any gender with insulin-treated diabetes, aged 18–75 years old, granted a class 1 (commercial licence), class 2 or LAPL (private licence) medical certificate to fly and currently participating within the EASA diabetes protocol. Pilots who were receiving either CSII, multiple daily injections (MDI) or a combination of insulin and oral glucose-lowering agents were included. Gender of participants was determined by self-reporting. The exclusion criteria were pilots outside of the stated age range or those not currently flying.

The protocol stipulated that measurement of SMBG obtained by finger-prick were made using an ISO 9000-certified device, awarded when the manufacturer meets the international set of standards of quality assurance and regulation requirements relating to its products. Pilots recorded measurements either 1 h before reporting for duty or 2 h before commencing a flight, then repeated them within 30 min before take-off, every hour during the flight, and within the 30 min before anticipated landing. Should a pilot experience symptoms that suggested a high or low blood glucose concentration then additional glucose testing was undertaken. Blood glucose values were verified by the co-pilot, read aloud to be captured by the cockpit voice recorder, and documented in the pilot’s logbook along with any action that was taken to correct out-of-range values.

Pilots underwent a medical review by an independent medical expert advising the national aviation authority every 6 months for commercial pilots or annually for private pilots. During these reviews, SMBG values documented in the logbook were checked against the pilot’s glucose meter recordings to ensure validity and protocol compliance. A full medical examination was performed, 6-monthly blood and urine results were reviewed, including HbA_1c_, lipid profile, renal function and urine microalbuminuria or albumin/creatinine ratio, and the results of annual cardiovascular and retinopathy screening were evaluated.

A traffic light system was devised as part of the protocol to aid the pilot’s interpretation of blood glucose values and determine acceptable and unacceptable glucose concentrations [14]. The acceptable range for blood glucose concentrations was 5.0–15.0 mmol/l (90–270 mg/dl) and was coded green. Values between 4.0–4.9 mmol/l (72–89 mg/dl) and 15.1–20.0 mmol/l (271–360 mg/dl) were coded amber to indicate caution and the potential need for intervention, and a glucose level of <4.0 mmol/l (<72 mg/dl) or >20.0 mmol/l (>360 mg/dl) was coded red and required immediate action.

#### Real-world observational study statistical analysis

Pre- and in-flight blood glucose values recorded by consenting pilots were validated and transferred to an Excel spreadsheet by two authors (GLG and DRJ) and then analysed using Microsoft Excel 2010 (Microsoft, USA) and SPSS statistics software version 28 (IBM, USA). Data are summarised either as mean ± SD or median (IQR or 95% CI). Due to the nature of this retrospective observational study, relationships are reported as associations only. Given that the variables being analysed were found not to follow a normal distribution (using Shapiro–Wilks test for normality), the Friedman test was used to test for significant difference in median values across time. When the null hypothesis of no difference between median SMBG values was rejected, the Bonferroni post hoc test was used to identify the timepoints where the significant differences were observed. Statistically significant results were those for which the *p* value was <0.05.

## Results

### Flight simulation study

**Fig. 3:**
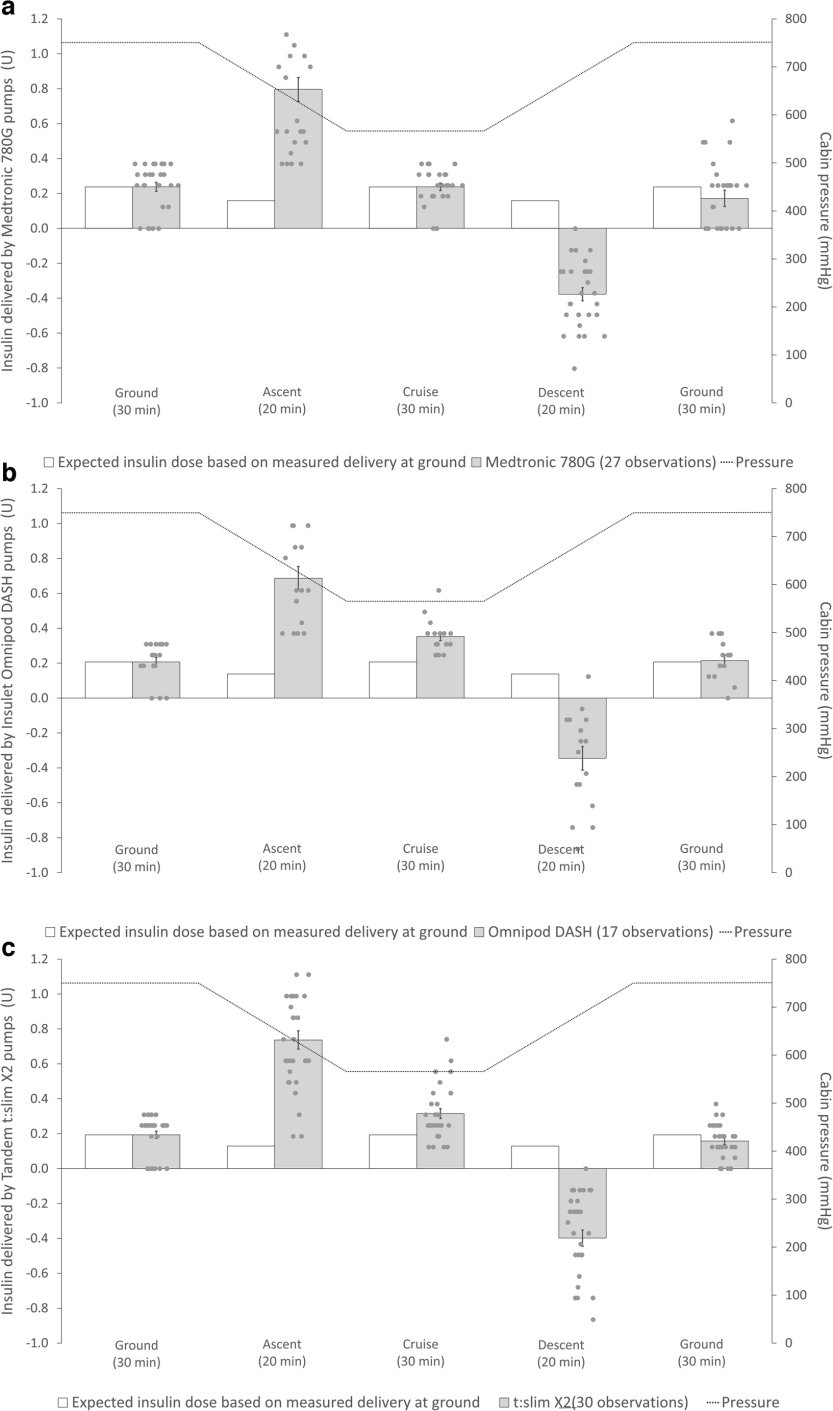
Illustration of the insulin delivery of each insulin pump by manufacturer: Medtronic 780G pump (**a**); Omnipod DASH pump (**b**); and Tandem t:slim X2 pump (**c**). The performance of each insulin pump brand with the phase of standard simulated flight protocol is shown, with grey data representing pump measurements during flight protocol, white representing measurements at baseline, and dotted line representing the cabin pressure during flight protocol. Medtronic 780G pumps, 27 observations; Omnipod DASH pumps, 17 observations; and Tandem t:slim X2 pumps, 30 observations

Seventy-four insulin pump datasets were analysed, and only four data points were discarded due to leakage. The amounts of insulin delivered by infusion are demonstrated as aggregated (Fig. 1b) and as individual pump models (Fig. 3a–c). Infused volume significantly increased above the pre-determined rate during ascent, remained stable during the cruise, and was significantly below the pre-determined infusion rate during descent. The changes in insulin delivery during the simulated flight were compared with the measured performance of 0.6 U/h at ground level (Table 1). The infused volume during standard flight protocol showed statistically significant differences during both ascent and descent when compared with other phases of flight. During ascent, insulin pumps delivered an additional 0.60 U (*p*<0.001) and descent delivered −0.51 U (*p*<0.001) of insulin compared with delivery at ground level.

**Table 1:** The infusion rate for each phase during the simulated flights when pumps were set to deliver at 0.6 U/h

Altitude/phase	No. of complete observations^a^	Measured insulin delivery (U)	Maximum insulin over-/under-delivered during phase (U)^b^
Ground (30 min)	74	0.21 ± 0.015	0.00
Ascent (20 min)	74	0.75 ± 0.040	0.60
Cruise (30 min)	74	0.30 ± 0.017	0.08
Descent (20 min)	74	−0.38 ± 0.029	−0.51
Ground (30 min)	74	0.18 ± 0.020	−0.04

Insulin delivery is presented as mean ± SEM

^a^A total of four sets of observations were discarded due to leakage

^b^Compared against the measured dose delivered at ground level and accounts for the time duration; over-delivery is represented as a positive value and under-delivery is represented as a negative value

Insulin pumps were not used during the rapid decompression sequence, only infusion sets. During rapid decompression, dyed fluid and bubbles were expelled from the top of the glass capillary tube, equivalent to a mean delivery of 5.6 U of insulin.

### Real-world observational study

Data from 49 pilots recorded over the 7.5-year study period were collated. Seven pilots received insulin by CSII during the study period. Two of these pilots were on conventional MDI therapy for part of the study before changing to CSII. Their demographic data were included in the CSII group and only their SMBGs recorded while receiving CSII were analysed.

#### Demographic data

Demographic data for all pilots are shown in Table 2. All seven pilots using CSII were male and had type 1 diabetes; four of them held a class 1 commercial licence. Their median age was 34.7 years (IQR 30.0–36.5), and the median diabetes duration was 12.2 years (IQR 7.1–27.1). The 42 pilots on MDI therapy were older, with a median age of 47.0 years (IQR 37.0–57.5), but with a similar duration of diabetes. Pre-certification mean HbA_1c_ was higher in the pilots using CSII (59.4 ± 13.2 mmol/mol [7.6 ± 1.2%]) than in pilots on MDI therapy (54.3 ± 9.1 mmol/mol [7.1 ± 0.8%]) but mean post-certification HbA_1c_ was similar in both groups (54.9 ± 8.3 mmol/mol [7.2 ± 0.8%] and 55.1 ± 9.9 mmol/mol [7.2 ± 0.9%], respectively). The mean follow-up period after licence certification was identical in both groups at 4.3 years. Using a paired *t* test, no statistically significant difference was found between the pre-certification HbA_1c_ and post-certification HbA_1c_ in either pilot group (*p*=0.231 in the CSII group and *p*=0.524 in the MDI group). eGFR was >60 ml/min per 1.73 m^2^ in all participants throughout the study and no pilot had a hypoglycaemic episode requiring third-party intervention in the 5 years preceding the study or during the study period.

**Table 2:** A comparison of demographic characteristics of pilots using CSII and pilots using MDI

Characteristic	Pilots using CSII (*n*=7)	Pilots using MDI (*n*=42)
Age, years	34.7 (30.0–36.5)	47.0 (37.0–57.5)
Gender		
Male	7 (100)	40 (95)
Female	0 (0)	2 (5)
Pilot certificate class		
Class 1	4 (57)	26 (62)
Class 2	3 (43)	16 (38)
Type of diabetes		
Type 1	7 (100)	34 (81)
Type 2	0 (0)	8 (19)
Duration of diabetes, years	12.2 (7.1–27.1)	10.9 (7.3–14.7)
Body weight, kg	90.0 (78.5–90.6)	81.0 (75.2–92.1)
Type of insulin		
Rapid-acting insulin (NovoRapid/Apidra/Aspart)	7 (100)	40 (95)
Long-acting insulin (Lantus/Levemir)	0 (0)	42 (100)
Adjuvant glucose-lowering therapy		
Biguanide (metformin)	1 (14)	9 (21)
Thiazolidinedione (pioglitazone)	0 (0)	2 (5)
Sulfonylurea	0 (0)	0 (0)
DPP-4 inhibitor	0 (0)	0 (0)
SGLT2 Inhibitor (empagliflozin)	0 (0)	1 (2)
GLP-1 agonist (exenatide/liraglutide)	0 (0)	3 (7)
Pre-certificate HbA_1c_, mmol/l	59.4 ± 13.2	54.3 ± 9.1
Pre-certificate HbA_1c_, %	7.6 ± 1.2	7.1 ± 0.8
Most recent HbA_1c_ (4.3 years post-certification), mmol/mol	54.9 ± 8.3	55.1 ± 9.9
Most recent HbA_1c_ (4.3 years post-certification), %	7.2 ± 0.8	7.2 ± 0.9
Presence of retinopathy		
No retinopathy	6 (86)	30 (71)
Background retinopathy	1 (14)	11 (26)
Retinopathy/maculopathy	0 (0)	1 (2)
Use of interstitial glucose-monitoring device		
None	0 (0)	29 (69)
Intermittently scanned glucose monitoring	4 (57)	8 (19)
Real-time CGM	3 (43)	5 (12)

Results are presented as *n* (%), median (IQR) or mean ± SD

DPP-4, dipeptidyl peptidase-4 inhibitor; GLP-1, glucagon-like peptide-1; SGLT2, sodium–glucose cotransporter 2

#### Blood glucose data for pilots using CSII

Table 3 compares all pre- and in-flight SMBG values recorded by each pilot group. A total of 4656 SMBG values were recorded during duty periods by the pilots using CSII. Of these, 4623 values (99.3%) were within the desirable green range, and only 33 (0.7%) values were outside of this range. The out-of-range values included 16 (0.3%) readings within the low amber range, 16 (0.3%) readings within the high amber range and one (0.02%) value in the low red range. No values were recorded in the high red range. More than two-thirds (69.7%) of the out-of-range values, including the single low red reading, were recorded in the pre-flight period. Ten (30.3%) out-of-range values were recorded in-flight with the lowest recorded value being 4.3 mmol/l.

**Table 3:** Total, pre-flight and in-flight capillary blood glucose measurements categorised according to the traffic light stratification system comparing pilots using CSII and pilots receiving MDI

Traffic light range	Pilots using CSII (*n*=7)	Pilots using MDI (*n*=42)
Total number of measurements	4656	33,965
Low red range (<4.0 mmol/l)		
Total	1 (0.02)	47 (0.1)
Pre-flight	1 (0.02)	33 (0.1)
In-flight	0 (0)	14 (0.04)
Low amber range (4.0–4.9 mmol/l)		
Total	16 (0.3)	534 (1.6)
Pre-flight	13 (0.3)	278 (0.8)
In-flight	3 (0.1)	256 (0.8)
Green range (5.0–15.0 mmol/l)		
Total	4623 (99.3)	33,106 (97.5)
Pre-flight	2112 (45.4)	13,806 (40.6)
In-flight	2511 (53.9)	19,300 (56.8)
High amber range (15.1–20.0 mmol/l)		
Total	16 (0.3)	272 (0.8)
Pre-flight	9 (0.2)	110 (0.3)
In-flight	7 (0.2)	162 (0.5)
High red range (>20.0 mmol/l)		
Total	0 (0.0)	6 (0.02)
Pre-flight	0 (0.0)	4 (0.01)
In-flight	0 (0.0)	2 (0.01)
Total out-of-range	33 (0.7)	859 (2.5)
Total amber range		
Total	32 (0.7)	806 (2.4)
Pre-flight	22 (0.5)	388 (1.1)
In-flight	10 (0.2)	418 (1.2)
Total red range		
Total	1 (0.02)	53 (0.2)
Pre-flight	1 (0.02)	37 (0.1)
In-flight	0 (0.0)	16 (0.1)
Total out-of-range		
Total	33 (0.7)	859 (2.5)
Pre-flight	23 (0.5)	425 (1.3)
In-flight	10 (0.2)	434 (1.3)

Data are expressed as *n* (%)

#### Blood glucose data for pilots using MDI

The 42 pilots on MDI therapy recorded 33,965 SMBG readings during the study period with 33,106 (97.5%) values in the acceptable green range, 806 (2.4%) values within the amber range, and 53 (0.2%) values recorded within the red range (Table 3). Two-thirds (66.3%) of the amber range readings were within the low amber range, with 47.9% reported in-flight. Of the 53 red range readings, 47 (88.7%) were within the low red, hypoglycaemic range, and the majority (*n*=33; 70.2%) of these occurred in the pre-flight period. Only 14 low red values were recorded in flight by pilots on MDI therapy. The lowest in-flight SMBG value recorded was 3.1 mmol/l. Six values were recorded in the high red range, two of which occurred in flight. The highest in-flight value was 21.1 mmol/l.

#### CSII out-of-range data

Sub-analysis of the three in-flight low amber range values recorded by pilots using CSII found that all had occurred within the second hour of three different flights and all three were reported by the same pilot. On each occasion there was a fall in glucose concentration from the previously recorded value: 12.3 mmol/l to 4.5 mmol/; 7.3 mmol/l to 4.3 mmol/l; and 10.6 mmol/l to 4.7 mmol/l. The time interval between readings was approximately 1 h on each occasion.

#### Blood glucose and flight patterns

Pilots receiving CSII undertook 1081 flights, accruing 2345 flying hours, throughout the study. Most flights (93.2%) were short-haul of under 3 h duration. Data analysis for pilots receiving CSII are displayed in electronic supplementary material (ESM) Tables 1, 2. The Friedman test showed no statistically significant difference between the median SMBG values recorded 30 min before take-off and the median SMBG values recorded at each hour in flight for flights with a duration of less than 60 min and over 180 min. For flights with a duration of between 61–120 min and 121–180 min, a statistically significant difference was observed between two median SMBG values, *p*=0.007 and *p*=0.018, respectively. Using Bonferroni post hoc analysis, a statistically significant difference was identified between the median SMBG values recorded <30 min before take-off (8.6 mmol/l) and <30 min before landing (8.2 mmol/l) for flights between 61–120 min, *p*=0.014. For flights with a duration of 121–180 min, Bonferroni post hoc analysis found no significant difference.

**Fig. 4:**
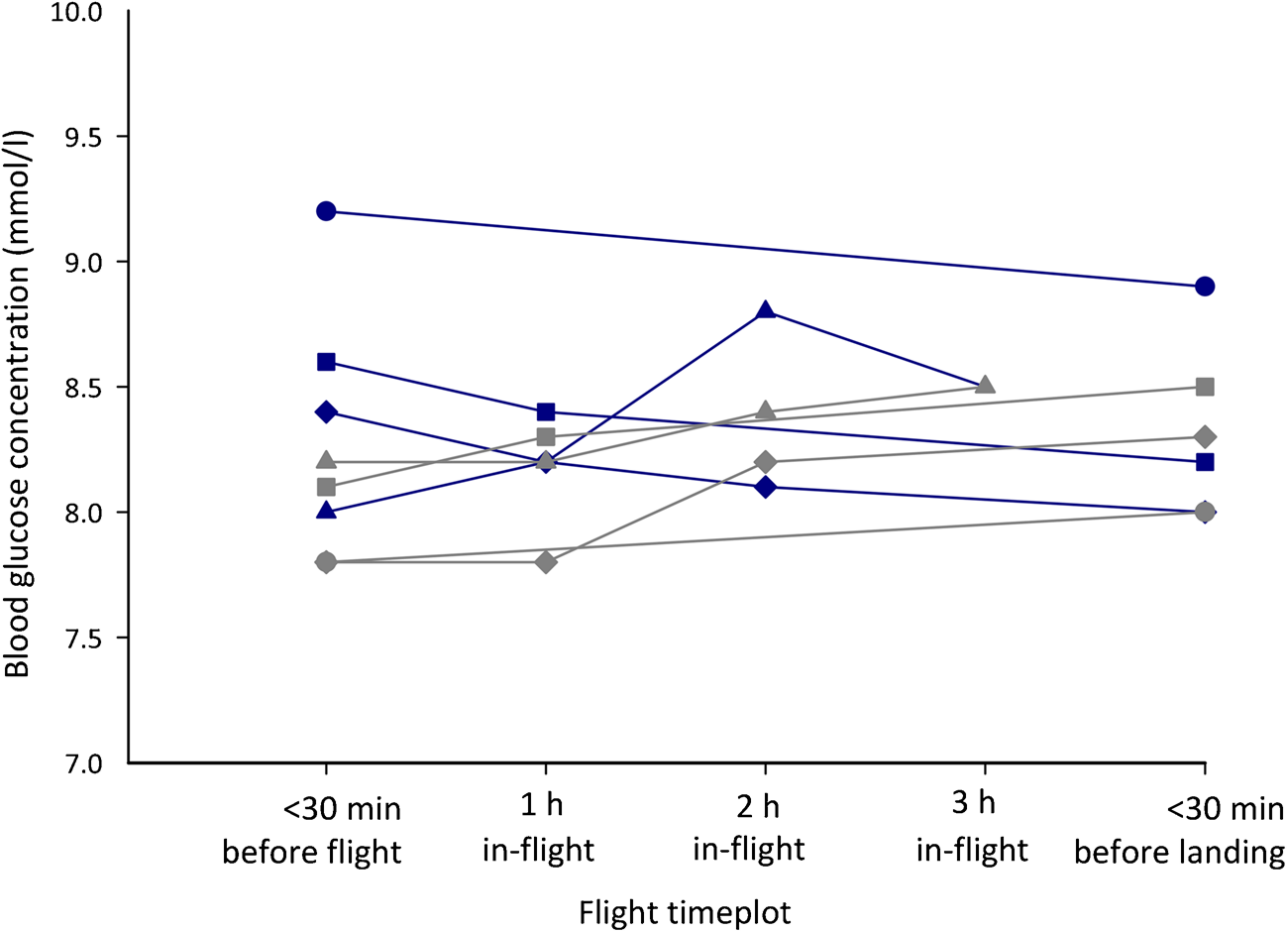
Line graph displaying the median SMBG values for pilots using CSII (blue symbols) compared with pilots receiving MDI therapy (grey symbols) recorded within 30 min before take-off and at each hourly timepoint in-flight for flight durations of 0–60 min (circles), 61–120 min (squares), 121–180 min (diamonds) and >180 min (triangles)

When analysing data for pilots on MDI therapy, both the Friedman test and Bonferroni post hoc analysis found numerous statistically significant differences in the median SMBG values when comparing timepoints recorded in all flight durations analysed over 60 min. Data for pilots on MDI therapy are displayed in ESM Tables 3, 4. Figure 4 shows the median SMBG values for pilots using CSII compared with pilots on MDI therapy that were recorded within 30 min before take-off and at each hourly timepoint in-flight per flight duration.

**Fig. 5:**
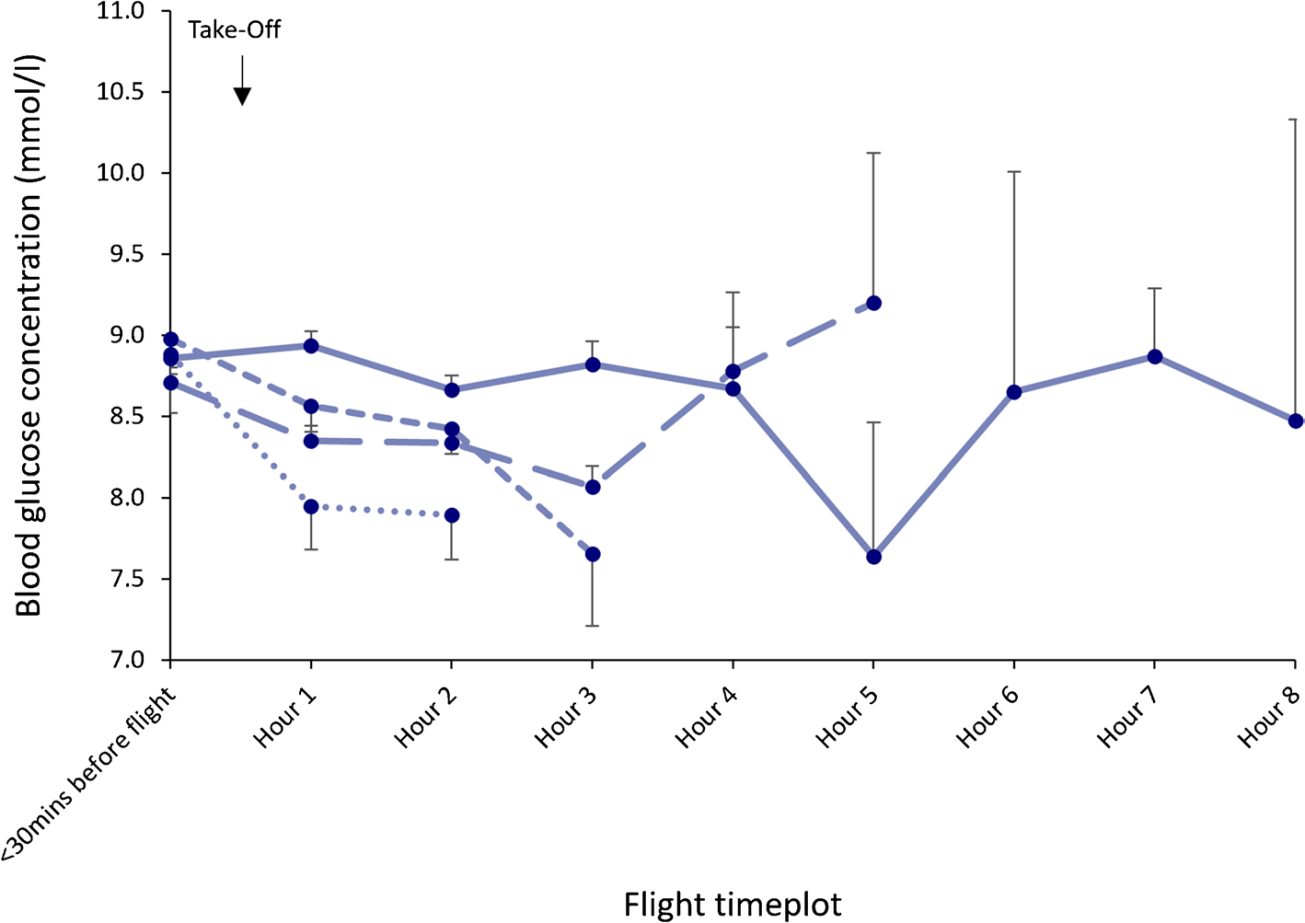
SMBG values for pilots using CSII according to hour of flight and for each subsequent flight performed within the same day. Solid line, flight 1; wide-dashed line, flight 2; narrow-dashed line, flight 3; dotted line, flight 4

Pilots frequently performed two or more flights on the same day. Figure 5 displays the mean SMBG value for all pilots using insulin pumps according to the hour of flight for each flight performed within a single day. In pilots using CSII, just over half (51.4%) of the SMBG values analysed were recorded during the first flight duty period. A further 38.8% of the SMBG values were recorded during the second flight and 9.6% were recorded during the third, fourth or fifth consecutive flight of the day. Of the 33 out-of-range values recorded by pilots using CSII, 30 (90.9%), including all ten in-flight out-of-range values, were recorded during the duty period for the first flight. Two low amber range values were recorded 30 min before take-off for the second flight and one high amber range value was recorded 30 min before take-off for the third flight of the day. No out-of-range values were reported pre- or in-flight during the fourth or fifth flights of the day. There was no evidence that repeated flights and repeat exposure to ambient pressure changes affected glycaemic control.

## Discussion

With an increasing number of pilots being granted medical certification to fly, the rising prevalence of diabetes globally and the growing application of technological devices in the management of both type 1 and type 2 diabetes, it is imperative to understand the effects of ambient pressure changes on such devices and the potential clinical manifestations that may ensue.

This is the first full-scale simulation of flight-associated atmospheric changes using a large decompression chamber to accurately simulate the cabin environment for modern insulin pumps and patch pumps. Our findings agree with earlier work in first-generation pumps in smaller pressure vessels [6, 7, 10, 15]. The present flight simulations have demonstrated that ambient pressure changes experienced during normal commercial flight cause unintended, but predictable, changes in insulin delivery. This equated to 0.60 U of excess insulin in response to a decrease in pressure replicating a 20 min ascent compared with the performance of pumps at ground level, and under-delivery of 0.51 U during an increase in pressure simulating a descent. This was likely attributable to air bubble formation–reabsorption and the respective expansion–contraction, as described by Henry’s and Boyle’s laws [8, 9]. This was most apparent during the violent expulsion of fluid and bubbles during rapid pressure changes, which resulted in fluid delivery equivalent to 5.6 U of excess insulin, far more than observed during the standard gradual ascent and descent.

When ambient pressure decreases, gas is released from liquids at an amount that is inversely proportional to the pressure change [7, 8]. The gases already dissolved within the insulin solution would be released during ascent, where bubbles form and displace insulin, causing excess delivery. While the volume of released gas is determined by Henry’s law constant, which depends on the mixture and solubility of the dissolved gases, any bubbles formed will expand by ~36% according to Boyle’s law [9, 15]. In contrast, bubbles re-dissolve with increasing pressures during descent and the potential space would draw the fluid back from the infusion set and cannula, leaving parts of the infusion line empty.

To understand the net effects of the observed delivery changes during ascent-to-cruise and descent-from-cruise, other factors must also be considered. The type of insulin, the structure of the insulin molecule, or the inert substances added to the insulin to maintain its integrity have been suggested to either contribute to or protect against air bubble formation in an insulin pump [16]. As such, the magnitude of degasification may differ with different insulin formulations. Another factor is variation in the temperature of the insulin solution. When the pump cartridge is refilled with cold insulin that has been stored in a fridge and subsequently warms by contact with the user’s body, or for example when a person leaves an air-conditioned environment in a hot climate, air bubbles gradually appear within the pump cartridge and infusion set. Because the bubbles appear gradually as the insulin warms, they would not be visible at the time the cartridge is refilled and the infusion set primed [15, 16]. Degasification of the insulin due to temperature rise would likely further contribute to the excess insulin delivery measured in the simulation study [16]. To control for the effects of temperature, the hypobaric chamber and insulin solutions were maintained at a constant temperature of 20°C. By eliminating temperature as a confounding factor, the effects of atmospheric pressure changes on insulin delivery by pumps can be better evaluated.

In keeping with these laws, our data represents absolute volume changes of these insulin pump devices, and the volume of excess delivery should be independent of the delivery rate of the device. As effervescence depends on fluid volume and expansion depends on pressure changes, insulin pumps with higher infusion rates are not expected to experience a greater change. Therefore, the full insulin cartridges that were used would represent the maximum anticipated change that any insulin pump user may experience.

These physical effects were most apparent during rapid decompression, where sudden and massive changes in pressure caused violent expansion from infusion sets alone. Unlike the gradual pressure changes used in the standard flight protocol simulation study, the sudden changes clearly demonstrated the formation and expansion of bubbles and degasification. Although rapid decompressions are rare, the dispelled volume of fluid was equivalent to 5.6 U of insulin. If a rapid decompression emergency occurs, pilots aim to descend to a flight altitude of approximately 3000 m (10,000 feet) to allow passengers to breathe unaided [17]. In this situation, CSII users, where possible, should try to pre-empt the effects of this over the next 2 h as insulin takes time to affect blood glucose. As such, users have up to 20 min post-event to anticipate and prevent hypoglycaemia by ingesting additional carbohydrates.

To explore the clinical implications of insulin pump over- and under-delivery in the real world, an observational study was performed. The aim was to determine whether there was any clinically significant fall in blood glucose concentration or episodes of hypoglycaemia associated with ambient pressure change within the aircraft during flight. The European experience in which SMBG concentrations are measured, recorded and independently verified on an hourly basis on every aircraft being flown by an insulin-treated pilot provided a means to evaluate insulin pumps in practice. This retrospective observational study analysed 4656 capillary blood glucose values recorded by pilots using CSII over 2345 h of flying across 1081 separate flights over a 7.5-year period. On each flight, the insulin pumps being used by the pilots were exposed to the same aircraft cabin pressure change.

The present study has observed that very few of the SMBG results recorded by the pilots using CSII fell outside the acceptable range and almost all of these, with one exception, were in the amber range. In comparison with pilots using CSII, pilots on MDI therapy would not be expected to experience any direct impact on their blood glucose concentration through changes in ambient cabin pressure. Despite this, pilots using CSII recorded a higher percentage of SMBG values within the desirable green range (99.3% vs 97.5%), fewer values in the low red range (0.02% vs 0.1%) and fewer in-flight out-of-range readings (0.2% vs 1.3%) when compared with those receiving MDI therapy. Furthermore, pilots using CSII maintained stricter glycaemic control while flying, with a narrower range of glucose concentrations. The lowest in-flight SMBG value recorded by CSII users was 4.3 mmol/l and the highest was 17.2 mmol/l as compared with 3.1 mmol/l and 21.1 mmol/l, respectively, in pilots on MDI therapy.

Median SMBG values for pilots using CSII were examined up to 180 min after take-off to capture any adverse effects from an excess insulin bolus. For flights lasting 61–120 min, a statistically significant fall in blood glucose concentration was observed when comparing the SMBG value recorded <30 min before take-off (8.6 mmol/l) and the pre-landing value (8.2 mmol/l). However, despite achieving statistical significance, the small difference in these median glucose concentrations is not of clinical importance. All pre- and in-flight median SMBG values across all timepoints and for all flight durations were within the acceptable green range (5.0–15.0 mmol/l) and no episodes of in-flight hypoglycaemia (<4.0 mmol/l) were experienced by any pilot using CSII.

On three separate occasions, an SMBG value in the low amber range was recorded by a pilot using CSII while flying. While this occurred less frequently in CSII users than in MDI users, who recorded 256 (0.8%) in-flight low amber range values, each value was reviewed individually in the context of insulin pump usage. All three were recorded during the second hour of the flight. One explanation is that they occurred because of the decrease in ambient pressure within the aircraft cabin, causing an over-delivery of insulin. However, these three low amber range SMBG values represent only 0.1% of all recorded in-flight SMBG values, equivalent to one low amber reading in every 360 flights made by a pilot with insulin-treated diabetes. Furthermore, the three values were recorded by the same pilot, supporting the likelihood that other factors relating to this individual’s management, rather than a reduction in cabin pressure, contributed to these three episodes of in-flight low blood glucose concentrations.

Factors specific to the individual pilot or to the CSII device can lead to degasification and air bubble formation and may have caused the low amber range values that were observed in the retrospective observational study. Pilot-specific factors include the degree of insulin sensitivity or resistance at a particular time of day, quality of overall glycaemic control, dietary intake, recent physical exercise, stress levels and concurrent illness. CSII-specific factors that can alter insulin dosing include the method used to refill the insulin cartridge, how careful the user is when expelling all air bubbles during priming of the infusion set, and the current volume of the insulin cartridge. One study reported the appearance of significantly more air bubbles in the infusion tubing shortly after the administration set had been changed and that the air bubble size was inversely correlated with the time since the infusion set change [18]. While the pre-set basal and bolus infusion rates do not influence the amount of insulin excess or deficit, CSII users who normally require very small doses of insulin may be at greater risk of clinical manifestations. The 0.60 U excess of insulin observed in the simulation study is unlikely to affect adults clinically but may explain the findings of previous case reports that document episodes of hypoglycaemia in children using CSII, who typically have lower insulin requirements [6, 7]. Although the present study did not collate data on these individual- or CSII-related confounding factors, it is plausible that one or more may have contributed to the low amber in-flight SMBG values observed.

Both the design and functioning of the insulin pump must meet approved standards set by the regulatory authorities. Pumps are built to function within limits when operating at the atmospheric pressures used in these studies. To meet these standards, the CSII design includes a built-in pressure equalisation system that allows for rapid equalisation of pressure between the pump cartridge and its environment, thus eliminating any pressure gradient during aircraft ascent and descent [19, 20]. As such, the pressure equalisation applies to the fluid without the effect of plunger movement. Contrarily, in vivo usage means that the CSII cartridge must overcome a counter pressure exerted by the subcutaneous tissue in order to deliver insulin. It has been suggested that the subcutaneous tissue in which the CSII cannula sits exerts a counter pressure at the tip of the needle, and this remains consistently greater than the ambient pressure regardless of external pressure changes [19]. Therefore, the pressure within the pump cartridge, which is influenced by the pre-set basal and bolus rates of the pump, will minimise the size of the air bubbles formed within the CSII system as gas is more compressible than fluid [16]. Consequently, the result is that of a closed pressure system that prevents unintentional excess insulin delivery during the usual pressure changes experienced during a flight [19]. While the differences in measured insulin dose delivered were 0.60 U and −0.51 U (426% and −367%, respectively, of expected delivery) in the simulation study, this greatly exceeds pump inaccuracies and variations measured in the published reports [21] and the true impact on delivery is likely much smaller in magnitude, or there is possibly no impact at all.

Any potential risk of clinical manifestations of insulin excess may be mitigated by taking precautions before and during flights, especially in individuals who may be more sensitive to small changes in insulin dose [7]. Where possible, pump users should adhere to manufacturer instructions, such as disconnecting infusion sets in unpressurised aircraft, or when rapid changes in altitude are expected [22–27]. Users may also consider disconnecting pumps before take-off and removing air bubbles before reconnecting when at cruising altitude. Pausing or switching to temporary delivery rates could also be considered. While opposite effects occur during ascent and descent, the real-time absorption is also affected by tissue back-pressures and the surface/volume ratio of boluses, hence, the delivery volume differs in magnitude and might not necessarily ‘cancel out’ across different flight phases [28]. Ultimately, insulin pump users should be aware of the theoretical increase in insulin delivery and risk of hypoglycaemia during ascent and adjust their glucose monitoring and carbohydrate ingestion appropriately [7].

A further important consideration is the concurrent use of interstitial glucose monitoring and hybrid closed-loop systems. Intermittently-scanned glucose monitoring (isCGM) or continuous glucose monitoring (CGM) systems provide near real-time data on blood glucose concentration. Many systems have an alarm that can warn users if the glucose level is rising or falling quickly to enable interventions to be made before dangerous levels are reached, thus facilitating stricter glycaemic control with fewer out-of-range values. Additionally, many modern insulin pumps can both receive and interpret CGM readings directly, creating a hybrid closed-loop system. In-built algorithms customised by the user to include details on insulin/carbohydrate ratios at different times of day enable the pump to interpret CGM readings in real-time and automatically calculate insulin correction doses, provided that an estimate of the carbohydrate content of meals is entered [29]. CSII systems also include an algorithm that interrupts the basal insulin infusion if the glucose concentration reaches a pre-determined level [30, 31]. These automated insulin delivery features help to increase CSII user safety by preventing both hypoglycaemia and hyperglycaemia through over- or under-delivery of insulin. This may be another reason why pilots using a combination of CSII and CGM did not experience any episodes of in-flight hypoglycaemia. The automated insulin delivery of hybrid closed-loop systems has received little assessment within the pressurised cabin environment and future work is required to explore this further.

During the 7.5 years of this retrospective study, interstitial glucose monitoring became more widely available and an increasing number of pilots across both groups began to adopt this technology. By the end of the study, a total of 20 pilots were using interstitial glucose monitoring. In the CSII group, 57% were using isCGM and 43% were using CGM compared with 19% and 11%, respectively, in the MDI group. However, while the use of isCGM and CGM systems is likely to have played a significant role in reducing the number of out-of-range values and hypoglycaemia events experienced by those pilots who used them, it is unlikely that the findings in this observational study can be attributed to the use of CGM alone.

### Limitations

Performing both in vitro and in vivo studies provided a controlled environment to determine the independent effects of atmospheric pressure changes on modern insulin pumps and their corresponding effects on glycaemic control in pilots in the real world. The use of Europe’s largest hypobaric chamber to simulate flight enabled confounding factors, including temperature and flight pattern, to be carefully controlled. However, it is not possible to mitigate against these confounding factors in real flight. Despite taking measures to control temperature during the simulation study, it was not possible to replicate the effects of aircraft vibrations that are experienced in flight. However, an earlier report that analysed bubble formation in insulin cartridges in response to vibrations found no correlation [15].

While every effort was made to prepare the cartridge and infusion set meticulously before use, and no bubbles were observed on visual inspection, the introduction of tiny amounts of gas could not be excluded. The use of digital microscopes or image analysis software [13] could have enhanced the ability to detect tiny bubbles and improve the accuracy of measuring insulin volume changes.

The simulation study evaluated insulin pumps in an open system, hence the resistance exerted by subcutaneous tissue, which affects both insulin delivery and absorption in real-time [28], was not recreated. Therefore, the measurements may overestimate the magnitude of the differences in insulin delivery. The findings in the observational study, which found no clinical manifestations of excess insulin delivery after ascent, would support this premise.

Only one infusion rate was used in the simulation study despite considerable variability in clinical practice among adult and paediatric CSII users. However, bubble formation is determined by fluid volume and is independent of pre-set infusion rates, therefore using a variety of pre-determined infusion rates would have been unlikely to have influenced the findings. Glycaemic control in pilots would also be affected by many factors including overall diabetes management during free-living, exercise, diet and illness, the details of which were not collated as part of the study.

All simulated flights in the hypobaric chamber consisted of a 20 min ascent, 30 min cruise and 20 min descent. No significant difference in insulin delivery was observed during the cruise phase because fluid and gas dynamics reached equilibrium within minutes. Therefore, the results obtained using this flight duration are expected to be representative of longer flights, where only the cruise phase is prolonged. Flight duration was not observed to affect glycaemic control in the real-world study.

In the real-world study reporting bias could occur, whereby pilots omit to record SMBG values that fall outside of the satisfactory, safe range. To mitigate against this, every blood glucose measurement performed while on flight duty had to be verified by the co-pilot, read aloud to be captured by the cockpit voice recorder and documented in the pilot’s logbook. SMBG values recorded in the logbook were later checked against the pilot’s glucometer recordings during the routine medical review to ensure the validity of data and compliance with the protocol.

Finally, all but two pilots who participated in this study were male, reflecting the longstanding gender imbalance within the profession. Unequal participant gender is not anticipated to have any impact on the generalisability of the study results as both men and women are exposed to the same potential complications of insulin pump use during aviation.

### Conclusion

This definitive study reports on the performance of modern insulin pumps in response to changing atmospheric pressures, and the clinical consequences on glycaemic control in insulin-treated pilots using CSII while flying. Flight simulation determined that the ascent and descent phases of flight can lead to unintended changes to insulin delivery. The real-world evaluation demonstrated that all insulin-treated pilots were assiduous and maintained excellent glycaemic control while flying. Pilots using CSII were not at a greater risk of glucose variability or episodes of hypoglycaemia during flight than pilots receiving MDI therapy and this retrospective study found no clinically significant falls in blood glucose concentration associated with the decrease in cabin pressure after aircraft take-off. As CSII enables many people with diabetes to achieve excellent metabolic control and improve clinical outcomes, its use remains effective within aviation and should be approved in the pressurised cabin environment of aircraft.

## Supplementary Information

Below is the link to the electronic supplementary material.

ESM Tables (PDF 135 KB)

## Data Availability

Authors agree to make data and materials supporting the results or analyses presented in their paper available upon reasonable request.

## Abbreviations

CGM: Continuous glucose monitoring
CSII: Continuous subcutaneous insulin infusion
EASA: European Union Aviation Safety Agency
LAPL: Light aircraft pilot licence
MDI: Multiple daily injections
SMBG: Self-monitored blood glucose
UK CAA: UK Civil Aviation Authority
