# Supplementary material for: Effects of atmospheric pressure change during flight on insulin pump delivery and glycaemic control of pilots with insulin-treated diabetes: an in vitro simulation and a retrospective observational real-world study
Source: Diabetologia. 2024 Nov 4;68(1):52–68. doi: 10.1007/s00125-024-06295-1 (PMC11663189; doi:10.1007/s00125-024-06295-1)
Supplement: Supplementary file 1 — ESM Tables (PDF 135 KB) [file 125_2024_6295_MOESM1_ESM.pdf]

Electronic supplementary material (ESM)

ESM Table 1: Statistical analysis on capillary blood glucose values for pilots receiving continuous subcutaneous insulin infusion (CSII) within 30 minutes before flight and for each time point in-flight.

Flight Duration (minutes)	Descriptive Statistics	Capillary Blood Glucose Concentration (mmol/L)					Number of Flights (n)	Friedmann Test p-value
		<30 mins Before Flight	Hour 1 In-Flight	Hour 2 In-Flight	Hour 3 In-Flight	<30 mins before Landing		
**0-60**	Median	9.2	-	-	-	8.9	163	0.115
	Mean (SD)	9.6 (2.4)	-	-	-	9.2 (2.4)		
	Minimum	3.9	-	-	-	5.0		
	Maximum	16.6	-	-	-	16.2		
**61-120**	Median	8.6	8.4	-	-	8.2	370	0.007
	Mean (SD)	8.8 (2.0)	8.7 (1.9)	-	-	8.4 (1.8)		
	Minimum	4.2	5.2	-	-	4.3		
	Maximum	16.0	14.8	-	-	14.4		
**121-180**	Median	8.4	8.2	8.1	-	8.0	331	0.018
	Mean (SD)	8.6 (1.7)	8.4 (1.8)	8.4 (1.8)	-	8.4 (1.8)		
	Minimum	4.4	5.1	5.1	-	5.1		
	Maximum	13.5	14.3	15.0	-	15.7		
**>180**	Median	8.0	8.2	8.8	8.5	-	67	0.550
	Mean (SD)	8.1 (1.5)	8.2 (1.5)	8.6 (2.0)	8.8 (2.3)	-		
	Minimum	5.1	5.1	4.7	5.4	-		
	Maximum	12.7	14.0	13.6	14.8	-		

ESM Table 2: Bonferroni Post Hoc p-values for median capillary blood glucose values between two specific timepoints pre- or in-flight for pilots receiving continuous subcutaneous insulin infusion (CSII).

Flight Duration (minutes)	Bonferroni Post Hoc p-values between Sample 1 - Sample 2					
	<30 Minutes Before Flight to Hour 1 In-Flight	<30 Minutes Before Flight to Hour 2 In-Flight	Hour 1 In-Flight to Hour 2 In-Flight	<30 Minutes Before flight to 30 Minutes Before Landing	Hour 1 in In-Flight to 30 Minutes Before Landing	Hour 2 In-Flight to 30 Minutes Before Landing
**61-120**	0.576	-	-	0.014	0.381	-
**121-180**	0.069	0.195	1.000	0.063	1.000	1.000

ESM Table 3: Statistical analysis on capillary blood glucose values for pilots receiving multiple daily injections (MDI) within 30 minutes before flight and for each time point in-flight.

Flight Duration (minutes)	Descriptive Statistics	Capillary Blood Glucose Concentration					Number of Flights (n)	Friedmann Test p-value
		<30 mins before flight	Hour 1 In-Flight	Hour 2 In-Flight	Hour 3 In-Flight	<30 mins before Landing		
**0-60**	Median	7.8	-	-	-	8.0	2660	0.530
	Mean (SD)	8.2 (2.2)	-	-	-	8.4 (2.2)		
	Minimum	3.2	-	-	-	3.6		
	Maximum	17.8	-	-	-	20.4		
**61-120**	Median	8.1	8.3	-	-	8.5	2232	0.006
	Mean (SD)	8.5 (2.4)	8.6 (2.3)	-	-	8.9 (2.3)		
	Minimum	3.6	4.2	-	-	3.9		
	Maximum	19.8	18.3	-	-	18.0		
**121-180**	Median	7.8	7.8	8.2	-	8.3	1287	<0.001
	Mean (SD)	8.2 (2.2)	8.1 (2.0)	8.5 (2.1)	-	8.6 (2.3)		
	Minimum	3.8	3.1	4.0	-	4.2		
	Maximum	22.0	16.9	18.5	-	21.1		
**>180**	Median	8.2	8.2	8.4	8.5	-	1189	<0.001
	Mean (SD)	8.4 (2.2)	8.5 (2.2)	8.7 (2.2)	8.8 (2.2)	-		
	Minimum	3.9	3.8	3.6	3.6	-		
	Maximum	18.1	18.7	19.3	17.5	-		

ESM Table 4: Bonferroni Post Hoc p-values for median capillary blood glucose values between two specific timepoints pre- or in-flight for pilots receiving multiple daily injections (MDI).

Flight duration (minutes)	Bonferroni Post Hoc p-values between Sample1 - Sample 2								
	<30 Minutes Before Flight to Hour 1 In-Flight	<30 Minutes Before Flight to Hour 2 In-Flight	<30 Minutes Before Flight to Hour 3 In-Flight	Hour 1 In-Flight to Hour 2 In-Flight	Hour 1 In-Flight to Hour 3 In-Flight	Hour 2 In-Flight to Hour 3 In-Flight	<30 Minutes Before flight to <30 Minutes Before Landing	Hour 1 In-Flight to 30 Minutes Before Landing	Hour 2 In-Flight to 30 Minutes Before Landing
**61-120**	1.000	-	-	-	-	-	0.015	0.044	-
**121-180**	0.796	0.016	-	0.000	-	-	0.002	0.000	1.000
**>180**	1.000	0.010	0.000	0.392	0.021	1.000	-	-	-
